# Factors impacting informed consent in cosmetic breast augmentation

**DOI:** 10.1016/j.breast.2023.02.007

**Published:** 2023-02-22

**Authors:** Stephen Whyte, Laura Bray, Martin Brumpton, Ho Fai Chan, Tim S. Peltz, Manisha Tamar, Uwe Dulleck, Dietmar W. Hutmacher

**Affiliations:** a School of Economics and Finance, Queensland University of Technology (QUT), 2 George St, Brisbane, QLD, 4001, Australia; b Centre for Behavioural Economics, Society & Technology (BEST), Queensland University of Technology (QUT), Brisbane, QLD, 4001, Australia; c Centre in Regenerative Medicine, Institute of Health and Biomedical Innovation, Queensland University of Technology (QUT), 60 Musk Avenue, Kelvin Grove, QLD, 4059, Australia; d ARC Training Centre for Cell and Tissue Engineering Technologies, Queensland University of Technology (QUT), Brisbane, QLD, 4059, Australia; e School of Mechanical, Medical and Process Engineering, Science and Engineering Faculty, Queensland University of Technology (QUT), 2 George St, Brisbane, QLD, 4001, Australia; f Behavioural Economics Team of the Australian Government (BETA), Department of Prime Minister & Cabinet, Canberra, Australia; g Surgical and Orthopaedic Research Laboratories, Prince of Wales Clinical School, University of New South Wales, Sydney, Australia; h Queensland University of Technology (QUT), 2 George St, Brisbane, QLD 4001, Australia; i ARC Training Centre in Additive Biomanufacturing, Queensland University of Technology, Brisbane, QLD, 4059, Australia; j ARC Training Centre for Multiscale 3D Imaging, Modelling and Manufacturing, Queensland University of Technology, Brisbane, QLD, 4059, Australia

**Keywords:** Cosmetic breast augmentation, Informed consent, Preferences, Revision surgery, Behavioural economics, Risk

## Abstract

**Background:**

For women who undergo cosmetic breast augmentation, their post-operative risk assessment may not match their pre-operative understanding of the involved risks and likelihood of revision surgeries. This may be due to the potential issues surrounding whether patients are being fully informed about all possible risks and related financial implications during the consent phases of patient/doctor consultation.

**Methods:**

To explore comprehension, risk preference, and perceptions of breast augmentation procedure, we conducted a recorded online experiment with 178 women (18–40 years) who received varying amounts of risk-related information from two experienced breast surgeons in a hypothetical first consultation scenario.

**Results:**

We find patient's age, self-rated health, income, education level, and *openness to experience* to be significant factors impacting initial breast augmentation risk preferences (before receiving any risk information). Further, more *emotionally stable* patients perceived greater breast augmentation risks, were less likely to recommend breast augmentation, and were more likely to acknowledge the likelihood for future revision surgery. After providing women with risk-related information we find increases in risk assessment in all treatment conditions, and that increased amounts of risk information do decrease women's willingness to recommend breast augmentation. But that increased risk information does not appear to increase women's assessment of the likelihood of future revision surgery. Finally, we find some participant individual differences (such as education level, having children, *conscientiousness* and *emotional stability*) appear to impact risk assessment post receiving risk information.

**Conclusion:**

Continuous improvement of the informed consent consultation process is vital to optimising patient outcomes efficiently and cost-effectively. Greater acknowledgement and emphasis on disclosure of related risks and financial burden when complications arise is also important. As such, future behavioural research is warranted into the factors impacting women's understanding both prior to and across the BA informed consent process.

## Highlights

• There is potentially an issue surrounding whether patients are being fully informed in cosmetic augmentation.
• We find women's age, self-rated health, income, education & openness to experience, are all factors impacting preference.
• After providing women with risk information we find increases in risk assessment in all treatment conditions.
• Education, children, conscientiousness, and emotional stability appear to impact risk assessment post risk information provision.

## 1 Introduction

Our study conducts a randomised video-recorded experiment varying the amount of risk related (informed consent) information 18-40-year-old women (*n* = 178) are communicated by two experienced Australian-based breast surgeons in a hypothetical “first consultation” scenario. We collect data on participant demographics, socio-sexuality inventory (SOI-R) and BIG 5 personality traits, as well as information regarding information retention, perceived risk, willingness to recommend cosmetic augmentation, and understanding the likelihood of future surgery, to explore factors that impact women's risk preferences both prior to, and after, the provision of cosmetic breast augmentation informed consent material.

## 2 Background

Complications after cosmetic breast augmentation (BA) are relatively common compared to other cosmetic plastic surgical procedures [[1], [2], [3]]. There is a growing consensus amongst plastic surgeons that during the consent phases of patient/doctor consultation, there exists a potential issue surrounding whether patients are being fully informed about the short-term and long-term risks, be it medical or financial [[3], [4], [5]]. While there are strict legal documents that explicitly point out all potential risks associated with BA surgery, these documents can be vague or insufficient in explicitly illustrating the likelihood of such events actually occurring for potential patients [6]. As such, potential patients may be irrationally attaching very small probabilities to negative surgical outcomes, relative to the actual probabilities of complications arising [7]. The translation of statistical likelihood for revision surgery into a real-life risk understanding by the patient is complicated and can even be difficult for medically trained experts to interpret and communicate [8]. Previous studies have suggested that consent forms be written to only a Year 6 level (11 years old) of language [9] because patients may not understand or would likely forget much of the information disclosed to them by their medical expert [10,11]. This is problematic when considering the long-term risks for patients who underwent cosmetic BA surgery at a relatively young age (mid ‘20s), as the likelihood of revision surgery over one's lifetime is close to 100%. Unfortunately, there is a void of quantitative behavioural and experimental research exploring how women process information relating to breast surgery [12,13] and how potential patients understand the risks associated with cosmetic breast augmentation.

To this end, we designed a randomised video-recorded experiment to examine what individual factors that contribute to women's initial risk assessment when considering a cosmetic augmentation procedure. We also explore whether explicit information provided by specialist breast surgeons in reality could increase or decrease risk preferences, understanding, and willingness to undergo cosmetic surgery.

## 3 Materials and methods

### 3.1 Data capture

Experimental design and data collection were conducted using the *z-tree* software,^1^ a software package used for developing and carrying out interactive online experiments (commonly used in the field of economics and psychology). Naturally, our study on cosmetic breast augmentation required our sample population to be women exclusively. All participants provided informed consent to participate, and all research was conducted in accordance with the approved QUT Human Research Ethics Committee protocol (approval no. 1900000908). Data were collected from 7–8 November 2020. All participants received a small flat rate remuneration amount ($40 AUD) for their participation time. Interactions with participants and survey data collection were conducted online and electronically.

### 3.2 Sample population

Participants were female members of the general population. Participants were only required to be between the ages of 18–40 with no medical history of cosmetic breast augmentation. No participant had any previous history or relationship with either surgeon involved in this study.

### 3.3 Survey data collected

Participants were first provided with a survey collecting a range of demographics such as age, height (cm), weight (kg), marital status, have offspring, education level, annual income, self-rated health (0–100 scale), and life satisfaction (0–100 scale), all variables commonly used in behavioural medicine and health research (e.g., Refs. [10,14,15]. Participants were provided a graphic of different breast sizes in ascending order (on a 7-point Likert scale), and then asked to choose the option that was most representative of their own breast size. In addition, we also measured the participant's sociosexual orientation and personality traits using the revised socio-sexual orientation inventory (SOI-R) test [16] and the Big 5 mini-marker personality test [17], respectively. The socio-sexuality inventory is a 9-item multidimensional self-reported survey scale used to measure individual differences in the tendency to have casual or uncommitted sexual relations (that is, sexual relationships without deeper commitment). SOI-R is a validated scale commonly used in social psychology [16]. The BIG 5 personality test measures a taxonomy of five personality traits (*openness to experience*, *conscientiousness*, *extraversion*, *agreeableness*, and *emotional stability*) and is useful for factor style (regression type) analysis exploring links between personality and behaviour. Higher numerical values for each trait indicate higher levels of the personality factor present. For example, more extroverted people have higher scores for “extraversion”. The BIG 5 personality test is a validated scale commonly used in personality psychology research (e.g., Refs. [18,19].

### 3.4 Experimental design

In the behavioural experiment, the participants were first provided with the demographic survey (see above description). Participants were then asked about their risk attitudes that reflect 1) their risk perception for the BA procedure (“*How risky (i.e., health complications) do you believe cosmetic breast augmentation is*?“), 2) their likelihood of recommending the BA procedure to a friend (“*Would you recommend breast augmentation to a friend?*“), and 3) their estimate of the likelihood of additional surgery after the BA procedure (“*How probable do you think additional surgery may be after a breast augmentation procedure?*“). All three questions were rated on a 0–100 scale. The participants were then shown a pre-recorded video monologue on informed consent risk information. After the video viewing, the participants were asked again about their risk attitudes towards the BA procedure using the three questions above.

Cosmetic augmentation for some women is a polarising topic. For this reason, and to maximise responses in our participant pool, the researchers did not ask women directly how likely “they are” to undergo a cosmetic breast procedure before or after the video. Instead, the study utilises a proxy for revealed preference, that of participants willingness to recommend the procedure to a friend. Further, to evaluate women's cognitive engagement in the video monologue, participants were asked a single question (post video) recording their recall on exactly how many specific risks the Doctor outlined and described to them. These data provide an important control and validity measure for both participants understanding and recall of the associated risks.

The pre-recorded informative videos consist of a monologue of a specialist breast surgeon (Royal Australian College of Surgeons (RACS) certified specialist Plastic Surgeon) emulating a hypothetical scenario in which the participant meets with the surgeon for the first consultation prior to an elective cosmetic breast augmentation procedure. The video monologue was uniform for all groups and included content taken from the required informed consent documents from St Luke's Private Hospital, Prince of Wales Private Hospital and East Sydney Private Hospital in Sydney, Australia, where such elective procedures are commonly undertaken (annually more than 300 and 400 times, respectively).

Our behavioural experiment consisted of a 2 × 2 design. Participants were randomly allocated into one of four conditions in which the gender of the surgeon (male vs female) and the amount of risk information presented in the video vary (baseline vs comprehensive risk). In the baseline risk condition, the risk information presented in the video monologue was taken from the procedure consent form classified as “short-run or minor risk” (baseline risk information). The baseline information covers six different short-run or minor risks associated with the BA procedure, including.

1. Bleeding during or after surgery
2. Infection from the surgical procedure
3. Interference with future mammograms
4. Seroma, a fluid collection around the implant
5. Change in nipple and breast sensation
6. Poor result

On the other hand, risk information categorised as “long run or higher risks” was also presented in the video monologue in the comprehensive-risk condition (comprehensive-risk) *in addition* to the baseline risk information. The following four longer-term or higher-risk outcomes from the elective procedure were presented after the baseline risk information.

1. Capsular contracture
2. Breast implant associated ALCL
3. Rupture and deflation of the implant
4. Secondary surgery and new financial costs.

Moreover, half of the participants assigned to the baseline and comprehensive risk conditions would receive the risk information presented by either a male or a female surgeon. For dependability of the experimental process, two currently practising Sydney-based certified plastic surgeons were engaged to present the information in the video recordings, Dr Jeremy Hunt^2^ and Dr Catherine Boorer.^3^ Both plastic surgeons were RACS accredited and experienced in performing the BA procedure and communicating the relevant supporting informed consent information to potential patients. Their role in the video was to explain to the participants the potential risks in the hypothetical scenario of the first cosmetic augmentation consultation.

In sum, we denote participant groups in the condition where they received only baseline risk information presented by a male or female surgeon as *male short* (*n* = 43) and *female short* (*n* = 40), respectively. The two participant groups which received additional risk information are referred to as *male long* (*n* = 45) and *female long* (*n* = 50), respectively. The transcripts of the monologue of the baseline (short-run risk only) and comprehensive (additional risk in the long-run) conditions are available in the supplementary material.

### 3.5 Analysis

Our analysis consists of two parts. The primary analysis concerns the role different individual factors play in the consent process for women towards BA procedure. In particular, using the ordinary least squares (OLS) regression, we examine how individual characteristics such as *age*, *height*, *weight*, *breast size*, *relationship status*, *offspring*, *education level*, *annual income*, *self-rated happiness*, *self-rated health*, *Big 5 personality traits*, and *socio-sexuality inventory* correlate with participants’ 1) risk preferences for cosmetic augmentation, 2) willingness to recommend the procedure, and 3) understanding of the likelihood of future surgery, measured *prior* to treatment exposure (i.e., video monologues of the BA procedure risk information). This analysis helps establishing the baseline of the roles of different individual factors play in the BA consent process for women.

For the second part of the analysis, we investigate the effect of *“risk/informed consent”* information (presented in the form of first consultation using the video monologue) on women's risk attitude towards a BA procedure based on our experimental design. To do this, we first utilise the paired *t-*test to compare the two set of participants' responses to the three questions relating to the comprehension, risk preference, and perceptions of BA procedure, recorded *before* and *after* viewing the video monologue with the risk information. Overall (i.e., pooling four treatments) and treatment specific (i.e., receiving information on *short-run* risk versus *long-run* risk and between male and female surgeon presenters) results are presented. For example, comparing the changes in response between the “*short*” and “*long*” groups allows us to identify whether the exposure to differing levels of risk information affect risk assessment differently. Lastly, we again apply OLS regression analysis to examine whether the individual factors (i.e., *age*, *education level*, *income*, *relationship status*, *offspring*) correlate with participants' risk attitudes towards BA procedure *after* receiving the risk information.

## 4 Results

### 4.1 Descriptive statistics

In Table 1, Table 2 we present descriptive statistics for our full sample population. Not included in our tables (as they are categorical variables) are the independent variables of relationship status and offspring. Rather, regarding relationship status, of *n* = 178 women in our sample, two-thirds were in some form of committed relationship (i.e., married, engaged, *de facto* relationship, *n* = 119; 66.85%) at the time of the study. Regarding offspring, approximately one-third stated they had at least one child (*n* = 67; 37.64%). Due to the UHREC requirements, participants did not have to complete any demographic question they did not wish to, resulting in our annual income variable only having *n* = 175 observations.

**Table 1:** Descriptive statistics – continuous variables.

*Variable*	*N*	Mean	Std. Dev.	Min	Max
**Age**	178	30.97	5.69	18	40
**Relationship**	178	0.67	0.47	0	1
**Offspring**	178	0.38	0.49	0	1
**Height (cm)**	178	165.92	7.54	146	187
**Weight (kg)**	178	78.09	25.13	42	179
**Breast size**	178	3.97	1.78	1	7
**Happiness**	178	75.22	18.12	3	100
**Health**	178	71.94	20.41	1	100
*Big 5 -Personality traits*					
**Extraversion**	178	4.68	1.12	2	7
**Agreeableness**	178	5.30	0.83	2.57	7
**Conscientiousness**	178	5.33	1.01	1.71	7
**Emotional stability**	178	4.57	1.01	1.57	6.71
**Openness**	178	4.23	0.84	1.85	6.14
**Socio-Sexuality** **Inventory (SOI-R)**	178	3.71	1.60	1	8.33

**Table 2:** Descriptive statistics – categorical variables.

	*N*	Percent
Income per annum		
- *Below $20,000*	30	17.14%
- *$20,001 - $40,000*	18	10.29%
- *$40,001 - $40,000*	29	16.57%
- *$60,001 - $40,000*	40	22.86%
- *$80,001 - $40,000*	34	19.43%
- *$100,001 - $120,000*	16	9.14%
- *$120,001 - $140,000*	*2*	1.14%
- *$140,000 and above*	6	3.43%
***Total***	*N* = 175	
**Education level**		
- *Grade 10*	2	1.12%
- *Grade 11*	8	4.49%
- *Grade 12*	30	16.85%
- *Technical/Pre-vocational*	44	24.72%
- *Undergraduate*	66	37.08%
- *Postgraduate*	28	15.73%
***Total***	*N* = 178	

#### 4.1.1 Multivariate analysis on BA risk attitudes prior to receiving risk information

In Table 3, we show the results of our multivariate analysis exploring the relationship between participant independent variables (individual differences) and the three key dependent variables measured *prior* to the risk information video treatment. The OLS regression results are presented in the three columns in Table 3 (models 1, 2, and 3).

**Table 3:** Factors impacting participant's BA procedure preferences *prior* to receiving risk information (baseline).

	(1) Perceived risk of procedure	(2) Likelihood to recommend procedure	(3) Likelihood of future surgery post procedure
**Age**	0.400 (0.398)	−1.175** (0.456)	−0.340 (0.446)
**Education**	−2.849 (1.909)	2.084 (2.212)	−5.286** (2.165)
**Income**	−1.427 (1.044)	3.191** (1.276)	−0.537 (1.331)
**Relationship**	−3.867 (4.648)	9.518* (5.576)	6.892 (5.709)
**Offspring**	−2.719 (4.558)	22.28*** (5.859)	−3.850 (5.355)
**Height (cm)**	0.464* (0.263)	−0.0513 (0.331)	−0.196 (0.309)
**Weight (kg)**	0.00493 (0.0910)	0.0882 (0.111)	0.0657 (0.128)
**Breast size**	−1.929 (1.213)	1.111 (1.674)	−1.817 (1.603)
**Happiness**	0.0715 (0.128)	−0.269 (0.166)	−0.00660 (0.166)
**Health**	0.272** (0.115)	0.0115 (0.153)	0.107 (0.141)
***Big 5 Personality traits***			
**Extraversion**	0.798 (1.682)	3.244 (2.191)	−0.103 (2.169)
**Agreeableness**	−0.444 (2.677)	−2.889 (3.383)	−2.676 (3.076)
**Conscientiousness**	−1.565 (2.047)	2.215 (2.842)	1.297 (2.363)
**Emotional Stability**	5.112*** (1.925)	−8.196*** (2.900)	5.776** (2.461)
**Openness**	−0.315 (2.536)	6.678** (3.228)	2.260 (3.165)
**SOI-R**	−1.840 (1.233)	3.772** (1.703)	0.654 (1.532)
**Constant**	−38.28 (45.93)	30.05 (54.90)	86.28* (50.53)
**N**	175	175	175
***R***^***2***^	0.1494	0.2525	0.0912
**Prob.** > ***F***	0.0020	0.0000	0.2060

Notes: OLS coefficient estimates. Standard errors (robust) in parentheses. *p < 0.10; **p < 0.05; ***p < 0.01.

We find women who self-rate as healthier placed higher perceived risk on cosmetic augmentation (*p* = 0.019). Likelihood to recommend the BA procedure is negatively correlated with participant's age (*p* = 0.011) but positively correlated with income (*p* = 0.013). Women who have offspring (compared with those that do not) are on average 22% more likely to recommend BA procedure (*p* < 0.01). Women with higher *openness* and those exhibiting higher socio-sexuality scores are also more likely to recommend (*p* = 0.04 and *p* = 0.028, respectively).

Across all three model specifications, we find strong statistically significant relationship between the personality trait *emotional stability* and BA risk attitudes. In our sample, women with higher scores in *emotional stability* perceive higher risks associated with cosmetic augmentation (*p* = 0.009), are less likely to recommend the procedure to a friend (*p* = 0.005), and state a higher likelihood of future medical revision post-surgery (*p* = 0.02).

### 4.2 Experimental results

**Fig. 1:**
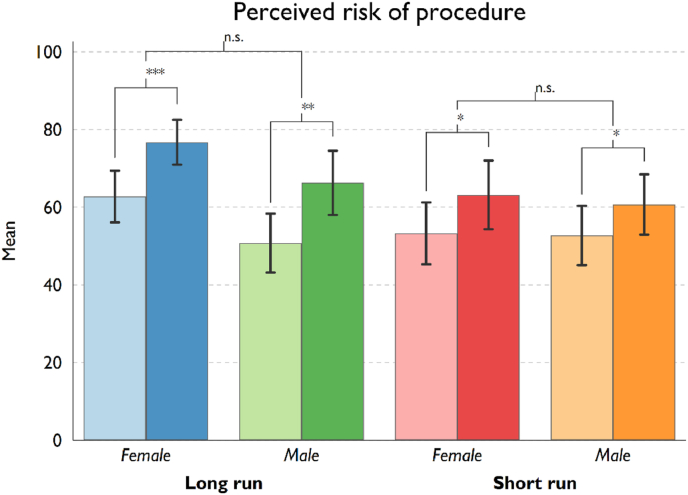
Perceived risk of procedure, pre and post surgeon video, differentiated by treatment conditions.

We begin our video experiment analysis with our primary dependent variable (perceived level of risk) relating to the hypothetical breast augmentation procedure. Our paired *t*-test analysis shows a statistically significant increase in the perceived riskiness of the breast augmentation procedure after participants are shown the informational video (overall *pre*-*post*-video difference equals 12 points, *p* < 0.001), for both participants receiving only baseline information (difference of 8.9 points, *p* = 0.0021) and those receiving additional long-run risk information (difference of 14.7 points *p* < 0.001). Fig. 1 provides a visual representation of the post-video increases in risk perception for each condition. We see that in all conditions, providing any level of risk information increases women's perceived risk of BA (see also Table 4, with *p*-values smaller than 0.05 in all treatment conditions).

**Table 4:** Effect of receiving risk information on BA Attitudes by treatment conditions.

Treatment	Presenter	Before	SD	After	SD	Diff.	*p*-value	*t*-stat.
**Perceived risk of procedure**								
Short-run risk	Female	53.25	24.87	63.18	27.66	9.93*	0.036	−2.18
	Male	52.72	24.83	60.69	24.93	7.95*	0.024	−2.34
Long-run risk	Female	62.74	23.40	76.72	20.37	13.98***	0.000	−4.70
	Male	50.76	25.27	66.27	27.48	15.51**	0.005	−2.94
**Likelihood to recommend procedure**								
Short-run risk	Female	33.00	36.26	26.50	33.76	−6.50†	0.079	1.80
	Male	34.44	36.40	28.24	32.95	−4.64	0.183	1.35
Long-run risk	Female	26.78	31.92	22.52	29.28	−4.26**	0.008	2.76
	Male	26.00	26.16	16.87	21.58	−9.13***	0.000	3.98
**Likelihood of future surgery post procedure**								
Short-run risk	Female	55.20	31.11	58.33	29.12	3.13	0.449	−0.76
	Male	51.42	31.07	53.74	29.61	1.33	0.691	−0.40
Long-run risk	Female	65.78	23.60	72.40	20.48	6.62*	0.028	−2.26
	Male	55.51	25.23	55.96	24.33	0.44	0.916	−0.11

Note: †*p* < 0.10; *p < 0.05; **p < 0.01; ***p < 0.001.

Nevertheless, we did not find the increase in risk perception among those who received additional long-run risk information to be statistically and significantly different from the baseline risk information group (mean difference = 12 points, *p* = 0.159). Furthermore, we did not observe a statistically significant gender effect (stemming from the difference in surgeon's gender) in the communication of the risk information (*p* = 0.939), either within the baseline risk information groups (*p* = 0.728) or the more comprehensive risk information groups (*p* = 0.796).

*Note: Lighter coloured bars (left bar within each pair) represent mean pre-video risk perceptions and darker coloured bars (right bar) represent average post-video risk perceptions. Statistical significance levels (two-tailed *t*-test): †*p* < 0.10; *p < 0.05; **p < 0.01; ***p < 0.001. The first level shows the pre-post video difference for each treatment condition. The second level indicates the statistical significance of the difference in the pre-post video change between subsamples by the gender of the surgeon. Error bars represent 95% confidence intervals.

**Fig. 2:**
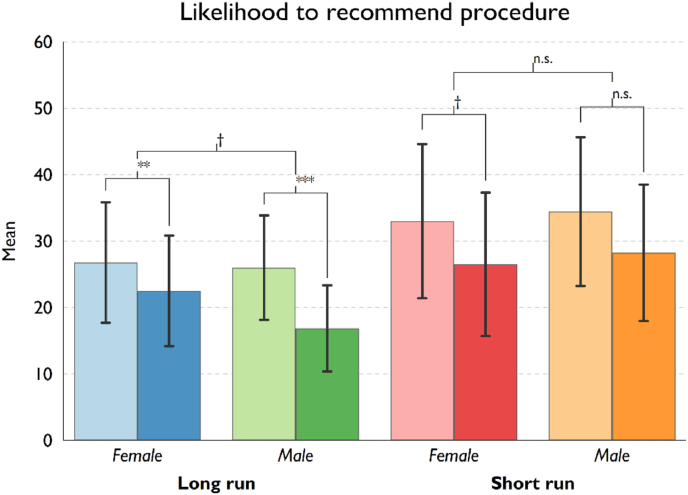
Likelihood to recommend procedure, pre and post surgeon video, differentiated by treatment conditions.

*Note: Lighter coloured bars (left bar within each pair) represent mean pre-video recommendation likelihood and darker coloured bars (right bar) represent average post-video recommendation likelihood. Statistical significance levels (two-tailed *t*-test): †*p* < 0.10; *p < 0.05; **p < 0.01; ***p < 0.001. The first level shows the pre-post video difference for each treatment condition. The second level indicates the statistical significance of the difference in the pre-post video change between subsamples by the gender of the surgeon. Error bars represent 95% confidence intervals.

We then analysed our second dependent variable, the willingness to recommend breast augmentation surgery to a friend, measured both before and after the video treatment. Overall, participants reported a lower tendency to recommend BA procedure to a friend upon receiving the risk information provided (overall reduction of 6.1 points, *p* < 0.01). However, we find that the decrease in recommendation likelihood seems to be larger for participants who received the long-run risk information (6.6 points decrease, *p* < 0.01) than those saw the video with only short-run risk (5.5 points decrease, *p* = 0.028). Moreover, in Fig. 2, we see that the difference in the pre- and post-video mean scores of those who received only the baseline risk information is not highly statistically significant (*p* = 0.079 for female surgeon condition; *p* = 0.183 for male surgeon condition, see also Table 4). On the other hand, we find that receiving additional long-run risk information seems to further reduce the likelihood of recommending BA procedure to a friend (*p* < 0.01 in both subsamples). Nevertheless, the difference in reduction between the two risk conditions is not statistically significantly different (*p* = 0.709). Similar to BA risk perception, we did not find the surgeon's gender mediates the video treatment effect (*p* = 0.531).

**Fig. 3:**
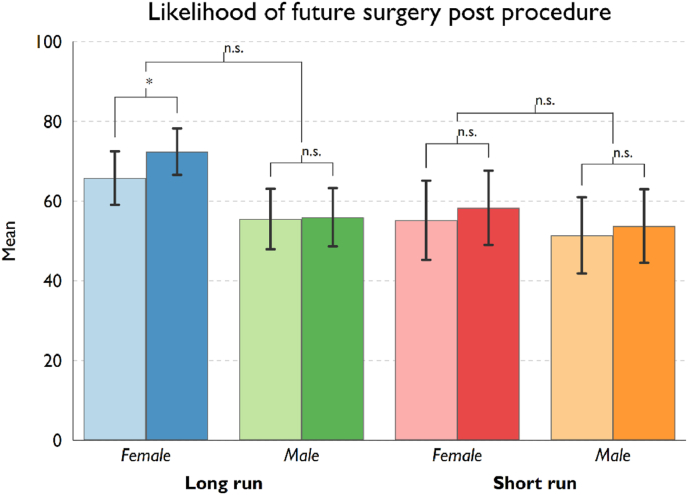
Likelihood of future revision surgery, pre and post surgeon video, differentiated by treatment.

*Note: Lighter coloured bars (left bar within each pair) represent the mean pre-video likelihood of future revision surgery and darker coloured bars (right bar) represent the average post-video likelihood of future revision surgery. Statistical significance levels (two-tailed *t*-test): †*p* < 0.10; *p < 0.05; **p < 0.01; ***p < 0.001. The first level shows the pre-post video difference for each treatment condition. The second level indicates the statistical significance of the difference in the pre-post video change between subsamples by the gender of the surgeon. Error bars represent 95% confidence intervals.

Finally, we analysed our third key variable of interest, participant's assessment of the likelihood of revision surgery, taken both before and after the video treatments (Fig. 3). With the exception of the *female long* treatment, where we observe an increase in the perception of the likelihood of revision survey (*p* = 0.028), we find no statistically significant results for change in women's estimation from before to after the risk information videos (overall mean pre-post video difference is only statistically significant at 10% level, *p* = 0.098; neither the short-run (*p* = 0.400) nor long-run risk (*p* = 0.144) subsample report a statistically significant pre-post difference). Nor any gender effect based on the surgeon presenting the information (*p* = 0.531).

#### 4.2.1 Individual differences impacting BA attitudes after receiving risk information

Next, we examine what individual factors influence participant's risk attitudes towards the BA procedure *after* receiving risk information using OLS regressions. Importantly, we controlled participant's baseline BA procedure preferences measured before the video monologue treatment. By doing so, we can examine whether the individual factors could explain the change in BA preferences due to the video treatment that is uncorrelated with the participant's *prior* preferences. Results are presented in Table 5. Additionally, we report the OLS results for the two subsamples of participants receiving either the baseline risk information or the comprehensive risk information in Table S1 in the Supplementary Material. In these set of regressions, we controlled for the gender of the surgeon in the treatment video.

**Table 5:** Factors impacting participant BA procedure preferences *after* receiving risk information.

	(1) Perceived risk of procedure	(2) Likelihood to recommend procedure	(3) Likelihood of future surgery post procedure
**Age**	0.227 (0.328)	0.0744 (0.211)	−0.176 (0.333)
**Education**	−7.017*** (1.495)	0.182 (1.215)	−4.679*** (1.550)
**Income**	0.377 (0.877)	0.900 (0.827)	−0.793 (0.925)
**Relationship**	−4.301 (3.888)	0.919 (2.994)	−4.451 (3.771)
**Offspring**	−9.007** (4.274)	4.252 (2.951)	−6.022 (3.957)
**Height (cm)**	0.0312 (0.240)	−0.0608 (0.209)	0.295 (0.217)
**Weight (kg)**	−0.0274 (0.0804)	0.0195 (0.0600)	−0.000589 (0.0809)
**Breast size**	−1.698 (1.079)	0.347 (0.857)	−1.375 (0.998)
**Happiness**	0.109 (0.165)	0.0122 (0.111)	0.0114 (0.121)
**Health**	−0.135 (0.113)	0.0222 (0.0962)	0.0626 (0.110)
***Big 5 personality traits***			
**Extraversion**	−1.069 (1.538)	0.349 (1.089)	0.603 (1.587)
**Agreeableness**	2.209 (2.596)	0.235 (2.294)	−0.783 (2.216)
**Conscientiousness**	2.160 (2.142)	−0.374 (1.659)	−4.037** (1.751)
**Emotional Stability**	0.785 (2.678)	−3.661** (1.573)	1.346 (2.032)
**Openness**	0.598 (1.883)	−0.922 (1.956)	0.196 (1.995)
**SOI-R**	−1.288 (1.071)	0.811 (0.759)	−0.460 (1.036)
***Prior to video***			
**Perceived risk**	0.419*** (0.0721)		
**Likelihood to recommend**		0.695*** (0.0735)	
**Likelihood of future surgery**			0.558*** (0.0624)
**Constant**	58.76 (45.47)	15.37 (32.39)	31.16 (40.36)
**N**	174	174	174
***R***^***2***^	0.3265	0.6965	0.4711
**Prob.** > ***F***	0.0000	0.0000	0.0000

Notes: OLS coefficient estimates. Standard errors (robust) in parentheses. *p < 0.10; **p < 0.05; ***p < 0.01.

#### 4.2.2 Participant age

We find no statistically significant relationship between the participant's age and their risk preference *after* being exposed to the video treatment (*p* = 0.316, Table 5 mode 1), which is similar to the baseline findings from Table 3 (*p* = 0.490). Also, we did not find a statistically significant effect of *age* on the likelihood of future surgery post-procedure whether the BA risk information was given to the participant (*p* = 0.447) or not (*p* = 0.598). However, after exposure to the risk information videos, the negative correlation between *age* and likelihood of recommending the BA procedure (see Table 3) is no longer visible (*p* = 0.598). That is, receiving risk information appears to have reduced the willingness to recommend BA procedure disproportionately more for the younger than the older participants. Notably, the null age effect on post-treatment BA procedure preferences is similar across the two subsamples (baseline and comprehensive risk information, Table S1).

##### 4.2.2.1 Participant education and income level

Similar to the baseline results (Table 3), we find no relationship between women's education level and their willingness to recommend breast augmentation after receiving the risk information (*p* = 0.881), controlling for their baseline willingness and other factors. However, whilst education is not correlated with participant's baseline BA procedure risk preference, it is negatively correlated with their ‘risk informed’ BA risk preference. That is, we saw a disproportionately larger reduction in the willingness to recommend breast augmentation procedure after receiving the risk information for participants with lower levels of education (*p* < 0.01). This result is robust to analyzing the subsamples of participants receiving only baseline risk (*p* = 0.097) and comprehensive risk information (*p* = 0.001), with the relationship being more robust to the latter. Lastly, we again find a negative correlation between education and likelihood for future revision surgery post-procedure after the risk information being presented to the participants (*p* = 0.003). However, this negative relationship does not seem to apply to those receiving only short-run risk information (*p* = 0.787). Participant's income levels do not explain the post-video treatment changes to any of the three dependent variables (*p* > 0.1 for all models).

#### 4.2.3 Participant relationship status and offspring

We find that being in a committed relationship (i.e., married, engaged, *de facto* relationship) shows no significant impact on the change in any of the three BA preferences, after viewing the treatment video (*p* = 0.27, *p* = 0.759, and *p* = 0.24, respectively). On the other hand, women without offspring seem to be more impacted by receiving BA risk information in terms of their risk assessment of the BA surgery. Specifically, participants with no offspring increased their BA risk assessment by an additional 9 points (in contrast to their risk evaluation before the video treatment) compared to women with offspring (*p* = 0.037). Nevertheless, such effect is not precisely estimated using the two subsamples whilst the coefficients bare the expected sign. Moreover, offspring status does not seem to have any effect on the change in recommend cosmetic breast augmentation (*p* = 0.152) nor likelihood of undergoing any future additional surgery (*p* = 0.130).

#### 4.2.4 Participant Big 5 personality traits and socio-sexuality inventory (SOI-R)

Next, we conduct examine the relationship between personality (Big 5 mini-marker - *openness*, *conscientiousness*, *extraversion*, *agreeableness*, and *emotional stability* – Saucier 1994) and attitudes towards BA after receiving risk information. None of the five traits is statistically significant in explaining the change in women's risk preference assessment after watching the treatment videos (*p* = 0.488, *p* = 0.396, *p* = 0.315, *p* = 0.770, and *p* = 0.751, respectively). With regards to the likelihood to recommend breast augmentation, we find that *emotional stability* account for stronger negative change in the willingness to recommend BA surgery after receiving the risk information (*p* = 0.021). Nevertheless, the coefficients based on the two subsamples are not statistically significant (with the expected sign). Finally, we find that *conscientiousness*, rather than *emotional stability*, seem to account for the change in participant's evaluations of the need for future additional surgeries post-procedure after obtaining BA-related risk information (*p* = 0.022). Such effect also seems to be more robust for participants who received the more comprehensive risk information (*p* = 0.02). Lastly, we find that post-video monologues changes in BA procedure preferences are not correlated with participant's SOI-R scores (*p* > 0.1 in all models).

## 5 Discussion

To ensure informed consent in cosmetic breast augmentation surgery, surgeons must provide every prospective patient with information about all alternatives and their associated risks and trade-offs [20]. However, individual variations in the patients choosing to engage in a cosmetic BA procedure can be large, and the historical uniform consent processes may not adequately meet all patient's needs [11]. As such, our study identifies key individual differences that impact women's risk preferences (*self-rated health* and *emotional stability*), willingness to recommend BA (*age*, *having children*, and the personality traits of *emotional stability* and *openness*, and *socio-sexuality*), and understanding of the likelihood of future revision surgery (*emotional stability*).

Importantly, our study's experimental design shows that the provision of any amount of risk information in the informed consent consultation process results in women stating increased risk perception after exposure (see Fig. 1). Further, our study finds that the provision of more risk information (relating to the long-term effects of BA) does result in women showing a substantial decreased willingness to recommend BA (as a possible proxy for actual consumption) (see Fig. 2). Regarding women's perception of the very high likelihood of revision surgery post-procedure [2,3], our results do not show increased information plays any significant role in women's assessment of the risks (see Fig. 3).

Previous research has shown positive relationships between breast augmentation and demographic variables such as education level [21]. Our study shows that education levels are negatively correlated with risk perception post-video exposure for our sample. Education clearly plays a role in both health behaviours and health decision-making [22], and such findings are important as they indicate that information provided in the current consent process is understandable irrespective of education level and that women do in fact, perceive greater risks associated with the elective procedure after consultation provision.

Our findings on patient's recall of the risks outlined in their video monologues indicate that while risk perceptions are generally higher when more risks are introduced, patient's margin for error in terms of retention and explicit recall of said information also increases. From a practical standpoint, such findings suggest the need for more patient-tailored informed consent materials, not greater amounts of risk content. Material with greater (or less) risk information may increase (or decrease) prospective patient's cognitive engagement and retention. For groups that begin the consultation process with a prior knowledge base that produces a pre-conceived or fixed position, it may be a barrier to their understanding.

Variance in secondary sexual characteristics, such as women's breasts, certainly impact individuals' and societies' expectations and perspectives on women's attractiveness [23]. Research across multiple cultures has demonstrated men's preferences for firmer, medium to large breasts size [24]. Research on women's preferences has also shown breast size to be associated with more positive attributes, including being popular, sexually active, assertive, and confident, with smaller breast size being associated with depression and loneliness [21]. It is then novel that our study finds no statistical relationship between participant's own breast size and any of the key variables of interest but instead found that (prior to our video monologue treatments) women with higher socio-sexuality scores (SOI-R) being more likely to recommend a cosmetic BA procedure. Further research is needed into the intersection of women's own biology, willingness to engage plastic surgery, social-cultural factors impacting idealisations of body shape, image, and beauty, and the rapidly evolving sexual liberalism of the new millennia.

Arguably the most interesting finding of our study relates to our robust findings for the personality trait of *emotional stability*, and its strong statistical relationship with women's assessments of risk, willingness to recommend, and understanding of the likelihood of future revision surgery, prior to the administering of any BA relevant risk information. *Emotional stability* is a process by which one continuously strives for better emotional health both intra-personally and intra-psychically, through the development of perceptions, feelings and attitudes that help in understanding and navigating the conditions, circumstances, and realities of life [25]. If greater emotional stability can be viewed as a more balanced or composed disposition [26], then our findings raise concern for a potentially vulnerable sub-populations of patients in the BA consent process. Those being women with lower reported emotional stability, who would appear to perceive lower risks, be more likely to recommend a BA procedure and state a lower likelihood of future revision procedures being necessary.

The current study is not without limitations. Firstly, our sample population are self-selected, and a convenience sample is limited to women 18–40 years. Secondly, our experimental design and video monologues (even though presented by actual plastic surgeons) are hypothetical scenarios and not real world “patient-surgeon” interactions. Finally, in our study “willingness to recommend” is only a proxy for actual elective surgery consumption decisions by patients in the cosmetic surgery space.

Future research would do well to collect data from prospective patients at the point of the first consultation or prior to, and subsequently track both patient expectations and risk preference in relation to actual real-world outcomes post-procedure.

From the supply side, future research may also explore any relationship between the economic incentive that is the substantial financial benefit to surgeons and communication of any possible trade-off or reduction in risk-related information in consultation, as well as organisationally via social media marketing. It is important to consider any possible cognitive or behavioural bias associated with the credence good of elective/cosmetic medical procedures, particularly with such limited current behavioural research in this space [27,28].

The most common serious complication arising in cosmetic breast augmentation is capsular contracture, and while reported rates vary widely in published literature [3,29,30], research has shown it also to be the most frequently listed fear for women having undergone a procedure [29]. Conventional breast augmentation surgery being based on some sort of implantation of a non-resorbable breast implant is like other implant-based surgeries prone to a variety of complications due to the nature of implanting a “foreign body”. In this context it would be interesting to further evaluate patients' understanding of this specific procedure, which by definition is a temporary treatment. No non-absorbable breast implant lasts forever, and depending on the patient's age at implantation, some complications will make revision surgery necessary at one point in the patient's life. Future behavioural research would do well to explore patient's understanding and propensity for associated risks when revision surgeries are instead explained to patients as a necessity and inevitability.

Finally, the internet and social media are now widely embraced as marketing tool by plastic and cosmetic surgeons, as well as being an easily accessible source of information for potential patients [29]. But such cyber platforms may be problematic for surgeons and the informed consent process for potential patients via perpetuation of misconceptions or fears from the unqualified or general public [30]. Little is known about how the internet is changing women's knowledge base and risk preferences towards elective cosmetic surgery outside regulated settings. That there is such a growing cosmetic tourism industry (nationally (inter-state) and internationally) with non-specialist surgeons performing procedures [30] speaks to some women's risk preferences being far higher.

## 6 Conclusion

While cosmetic breast augmentation has been central to plastic surgeons' practises for more than 50 years, patient outcomes are still not ideal [2,31,32]. Research indicates that close to one in five women believe their pre-operative expectations did not match the experience and post-operative results [29]. While reported rates of silicone implant re-operation vary, multiple studies state rates greater than 25% at ten years [[33], [34], [35]]. Optimising patient outcomes requires clearly defining the overall process for patients [31] with the most efficient and cost-effective way being the continuous improvement of the informed consent consultation process. With greater acknowledgement and emphasis on disclosure of real and possible risks [32] and who should bear the financial burden when complications arise [3]. As such, future behavioural research is warranted into the factors that impact women's explicit understanding and risk preferences prior to and across the BA informed consent process.

## Footnotes

1 https://www.ztree.uzh.ch/en.html.

2 https://www.drjeremyhunt.com.au/.

3 http://catherineboorer.com.au/.

## References

[1] Sullivan S.R., Fletcher D.R., Isom C.D., Isik F.F. (2008). True incidence of all complications following immediate and delayed breast reconstruction. Plast Reconstr Surg.

[2] Khersonsky J., Spanknebel K., Rosenberg M., DeLuca-Pytell D., Palaia D., Bonanno P., Petro J. (2009). Cosmetic breast augmentation: long-term study of outcome and revision procedures in 56 patients over 4 decades. Am J Cosmet Surg.

[3] Miller G.S., Robinson S., Reid C.M., Hunter-Smith D.J. (2018). Cosmetic breast augmentation in Australia: a cost of complication study. Australasian Journal of Plastic Surgery.

[4] Zuckerman D., Abraham A. (2008). Teenagers and cosmetic surgery: focus on breast augmentation and liposuction. J Adolesc Health.

[5] Van Slyke A.C., Carr M., Carr N.J. (2018). Not all breast implants are equal: a 13-year review of implant longevity and reasons for explantation. Plast Reconstr Surg.

[6] Hanson M., Pitt D. (2017). Informed consent for surgery: risk discussion and documentation. Can J Surg.

[7] Kahneman D. (2011).

[8] Anderson O.A., Wearne I.M.J. (2007). Informed consent for elective surgery—what is best practice?. J R Soc Med.

[9] Sherlock A., Brownie S. (2014). Patients' recollection and understanding of informed consent: a literature review. ANZ J Surg.

[10] Chan Y., Irish J.C., Wood S.J., Rotstein L.E., Brown D.H., Gullane P.J., Lockwood G.A. (2002). Patient education and informed consent in head and neck surgery. Arch Otolaryngol Head Neck Surg.

[11] Falagas M.E., Korbila I.P., Giannopoulou K.P., Kondilis B.K., Peppas G. (2009). Informed consent: how much and what do patients understand?. Am J Surg.

[12] Whyte Stephen, Bray Laura, Chan Ho Fai, Chan Raymond J., Hunt Jeremy, Peltz Tim S. (2022). Cognitive bias and therapy choice in breast reconstruction surgery decision-making. Plast Reconstr Surg.

[13] Whyte S., Bray L.J., Chan H.F., Chan R.J., Hunt J., Peltz T.S., Dulleck U., Hutmacher D.W. (2022). Knowledge, consultation time, and choice in breast reconstruction. Br J Surg.

[14] Whyte S., Torgler B. (2016). Determinants of online sperm donor success: how women choose. Appl Econ Lett.

[15] Whyte S., Savage D.A., Torgler B. (2017). Online sperm donors: the impact of family, friends, personality and risk perception on behaviour. Reprod Biomed Online.

[16] Penke L., Asendorpf J.B. (2008). Beyond global sociosexual orientations: a more differentiated look at sociosexuality and its effects on courtship and romantic relationships. J Pers Soc Psychol.

[17] Saucier G. (1994). Mini-Markers: a brief version of Goldberg's unipolar Big-Five markers. J Pers Assess.

[18] Whyte S., Torgler B. (2017). Things change with age: educational assortment in online dating. Pers Indiv Differ.

[19] Chan H.F., Moon J.W., Savage D.A., Skali A., Torgler B., Whyte S. (2021). Can psychological traits explain mobility behavior during the COVID-19 pandemic?. Soc Psychol Personal Sci.

[20] Tebbetts J.B., Tebbetts T.B., Gorney M. (2002). An approach that integrates patient education and informed consent in breast augmentation. Plast Reconstr Surg.

[21] Figueroa-Haas C.L. (2007). Effect of breast augmentation mammoplasty on self-esteem and sexuality: a quantitative analysis. Plast Surg Nurs.

[22] Cutler D.M., Lleras-Muney A. (2006). https://www.nber.org/system/files/working_papers/w12352/w12352.pdf.

[23] Furnham A., Dias M., McClelland A. (1998). The role of body weight, waist-to-hip ratio, and breast size in judgments of female attractiveness. Sex Roles.

[24] Havlíček J., Třebický V., Valentova J.V., Kleisner K., Akoko R.M., Fialová J., Roberts S.C. (2017). Men's preferences for women's breast size and shape in four cultures. Evol Hum Behav.

[25] Smithson W.B. (1974).

[26] Chaturvedi M., Chander R. (2010). Development of emotional stability scale. Ind Psychiatr J.

[27] Smith H., Whyte S., Chan H.F., Kyle G., Lau E.T., Nissen L.M., Dulleck U. (2019). Pharmacist compliance with therapeutic guidelines on diagnosis and treatment provision. JAMA Netw Open.

[28] Whyte S., Bray L.J., Chan H.F., Chan R.J., Hunt J., Peltz T.S., Dulleck U., Hutmacher D.W. (2022). Exploring Surgeons', nurses' and patients' information seeking behaviour on medical innovations: the case of 3D printed biodegradable implants in breast reconstruction. Annals of Surgery Open.

[29] Walden J.L., Panagopoulous G., Shrader S.W. (2010). Contemporary decision making and perception in patients undergoing cosmetic breast augmentation. Aesthetic Surg J.

[30] Deva A.K., Cuss A., Magnusson M., Cooter R. (2019). The “Game of Implants”: a perspective on the crisis-prone history of breast implants. Aesthetic Surg J.

[31] Adams W.P. (2008). The process of breast augmentation: four sequential steps for optimizing outcomes for patients. Plast Reconstr Surg.

[32] Steve A.K., Temple-Oberle C., Yeung J.K., Lafreniere A.S., Harrop A.R. (2021). You helped create this, help me now”: a qualitative analysis of patients' concerns about breast implants and a proposed strategy for moving forward. Plast Reconstr Surg.

[33] Caplin D.A. (2014). Indications for the use of MemoryShape breast implants in aesthetic and reconstructive breast surgery: long-term clinical outcomes of shaped versus round silicone breast implants. Plast Reconstr Surg.

[34] Spear S.L., Murphy D.K. (2014). Natrelle round silicone breast implants: core study results at 10 years. Plast Reconstr Surg.

[35] Maxwell G.P., Van Natta B.W., Bengtson B.P., Murphy D.K. (2015). Ten-year results from the Natrelle 410 anatomical form-stable silicone breast implant core study. Aesthetic Surg J.

