# Supplementary material for: Factors impacting informed consent in cosmetic breast augmentation
Source: Breast. 2023 Feb 22;68:225–32. doi: 10.1016/j.breast.2023.02.007 (PMC9996440; doi:10.1016/j.breast.2023.02.007)
Supplement: Multimedia component 1 [file mmc1.docx]

# Supplementary material

**Monologue 1:** Baseline Group information: Short Run or Minor Risks

**Hello my name is Dr [Hunt/Boorer], and I’m a Plastic Surgeon based in Sydney at the Prince of Wales & St Luke’s hospitals.**

This is a short informed consent video designed to assist Surgeons in ensuring patients understanding of the benefits, risks, hazards and alternative treatments related to a specific planned surgical treatment. Today I’m going to talk with you about one of the procedures I often perform, cosmetic breast augmentation.

For the purposes of this video I’m going to talk to you as if you are my patient. As if you have made the decision to undergo a breast augmentation or implant procedure, and we are in the process of discussing all possible outcomes before you make your final decision and provide your written consent.

After the video the Queensland University of Technology research team are going to continue with their survey and ask you some more questions about what you thought of the video and the information that I provided.

Okay let’s begin.

You have the right, as a patient, to be informed about your condition and the recommended surgical, medical or diagnostic procedures that will be performed. The information I will now provide you will enable you to make an informed final decision. Informed consent communications are used to provide disclosure on possible risks and alternative forms of treatment. Any informed consent materials and communications should not be considered all-inclusive in defining other methods of care and possible risks encountered. Informed consent materials are not intended to define or serve as the standard of medical care. Standards of medical care are determined on the basis of all the facts involved in an individual case and are subject to change with scientific and technical advances.

So here are some of the possible risks involved in cosmetic breast augmentation.

**Short Run:**

Here are some of the possible short run risks involved:

**Possible Risks 1. – Bleeding during or after surgery**

- Should postoperative bleeding occur, it may require emergency treatment to drain accumulated blood or a blood transfusion. If your implants have been inserted through the armpit, an incision under the breast may be necessary for a large haematoma to be drained

**Possible Risks 2. – Infection from the surgical procedure**

- If your implants have been inserted through the armpit, an incision under the breast may be necessary for an implant to be removed

**Possible Risks 3. – Interference with future mammograms**

- An implant can interfere with the detection of early breast cancer because it may "hide" suspicious legions in the breast during an X-ray exam

**Possible Risks 4. – Seroma, a fluid collection around the implant**

- A fluid collection may occur around the implant and require drainage with a needle guided by Ultrasound. This may need to be performed more than once.

**Possible Risks 5. – Poor Result**

- There is the possibility of a poor result from the breast augmentation/Implant surgery. You may be disappointed with the results of surgery, visible deformities, visible asymmetry and unacceptable shape. You may be dissatisfied with the degree of improvement after surgery.

That completes the list of possible risks to a surgical breast augmentation procedure. Thank you for listening to our information briefing.

You will now be returned to the survey to answer some final questions.

**Monologue 2:** Comprehensive risk information Group information: Long Run or Higher Risks

**Hello my name is Dr [Hunt/Boorer], and I’m a Plastic Surgeon based in Sydney at the Prince of Wales & St Luke’s hospitals.**

This is a short informed consent video designed to assist Surgeons in ensuring patients understanding of the benefits, risks, hazards and alternative treatments related to a specific planned surgical treatment. Today I’m going to talk with you about one of the procedures I often perform, cosmetic breast augmentation.

For the purposes of this video I’m going to talk to you as if you are my patient. As if you have made the decision to undergo a breast augmentation or implant procedure, and we are in the process of discussing all possible outcomes before you make your final decision and provide your written consent.

After the video the Queensland University of Technology research team are going to continue with their survey and ask you some more questions about what you thought of the video and the information that I provided.

Okay let’s begin.

You have the right, as a patient, to be informed about your condition and the recommended surgical, medical or diagnostic procedures that will be performed. The information I will now provide you will enable you to make an informed final decision. Informed consent communications are used to provide disclosure on possible risks and alternative forms of treatment. Any informed consent materials and communications should not be considered all-inclusive in defining other methods of care and possible risks encountered. Informed consent materials are not intended to define or serve as the standard of medical care. Standards of medical care are determined on the basis of all the facts involved in an individual case and are subject to change with scientific and technical advances.

So here are some of the possible risks involved in cosmetic breast augmentation.

**In the short run:**

Here are some of the possible short run risks involved:

**Possible Risks 1. – Bleeding during or after surgery**

- Should postoperative bleeding occur, it may require emergency treatment to drain accumulated blood or a blood transfusion. If your implants have been inserted through the armpit, an incision under the breast may be necessary for a large haematoma to be drained

**Possible Risks 2. – Infection from the surgical procedure**

- If your implants have been inserted through the armpit, an incision under the breast may be necessary for an implant to be removed

**Possible Risks 3. – Interference with future mammograms**

- An implant can interfere with the detection of early breast cancer because it may "hide" suspicious legions in the breast during an X-ray exam.

**Possible Risks 4. – Seroma, a fluid collection around the implant**

- A fluid collection may occur around the implant and require drainage with a needle guided by Ultrasound. This may need to be performed more than once.

**Possible Risks 5. – Changes in nipple and breast sensation**

- Any surgery on the breast, including a biopsy or breast implant surgery, can result in increased or decreased sensation of the breast or nipple. This change can vary in degree and may be temporary or permanent. It may affect comfort with breast feeding or sexual response. Incision in the armpit can affect the feeling under the armpit, temporarily or permanently.

**Possible Risks 6. – Poor Result**

- There is the possibility of a poor result from the breast augmentation/Implant surgery. You may be disappointed with the results of surgery, visible deformities, visible asymmetry and unacceptable shape. You may be dissatisfied with the degree of improvement after surgery.

**In the long run:**

Here are some of the possible long run risks involved:

**Possible Long Run Risk 1. – Capsular contracture, a type of scar tissue that forms around the breast**

- The scar tissue that forms around the implant can tighten and squeeze the implant as a natural response to having any foreign object implanted in the body making it feel firm. It can cause varying degrees of discomfort and pain, and misshape the appearance of the breast. Reported capsular contracture rates vary, with some current research indicating that somewhere between 10.4% up to 40.4% of women need further medical treatment for complications in their first 3 years. That is as many as two out of every five women.

**Possible Long Run Risk 2. – Breast Implant Associated ALCL – Which is a type of Lymphoma**

- **BIA ALCL** is a cancer of lymphatic cells and a form of Non-Hodgkin's Lymphoma. It occurs in association with exposure to textured implants and takes an average of 7 -10 years after implant insertion before it develops.

**Possible Long Run Risk 3. – Rupture and deflation of the implant**

- The shell of a saline implant may rupture and this causes leakage of the saline into the tissue and deflation of the implant. This would require surgery and replacement of the implant. The rate of deflation of saline implants is approximately 10% after 5 years.

**Possible Long Run Risk 4. – Secondary surgery and new financial costs**

- Should you have any further surgery, the surgeons and hospital fees will be your responsibility, and may amount to more than you are spending on the current procedure. Subsequent alterations in the breast appearance may occur as a result of aging, weight gain or loss, sun exposure, or circumstances not related to the breast augmentation/implant surgery. Breast augmentation/Implant surgery does not arrest the aging process. Future surgery or treatment may be necessary to maintain the results of breast augmentation/Implant surgery.

That completes the list of both short and long risks to a surgical breast augmentation procedure. Thank you for listening to our information briefing.

You will now be returned to the survey to answer some final questions.

Table S1. Factors impacting participant BA procedure preferences *after* receiving baseline and comprehensive risk information

|  | *(1)* | *(2)* | *(3)* | *(4)* | *(5)* | *(6)* |
| --- | --- | --- | --- | --- | --- | --- |
|  | **Perceived risk of procedure** | | **Likelihood to recommend procedure** | | **Likelihood of future surgery post procedure** | |
|  | Long-run risk | Short-run risk | Long-run risk | Short-run risk | Long-run risk | Short-run risk |
| **Age** | 0.505 | 0.0185 | 0.134 | 0.121 | -0.140 | -0.613 |
|  | (0.361) | (0.594) | (0.224) | (0.568) | (0.405) | (0.573) |
| **Education** | -7.404^***^ | -6.067^*^ | 0.702 | -0.207 | -4.042^*^ | 1.037 |
|  | (2.105) | (3.606) | (1.085) | (3.370) | (2.198) | (3.812) |
| **Income** | 0.362 | 0.239 | 0.688 | 1.224 | -1.303 | -0.133 |
|  | (1.256) | (1.509) | (0.830) | (1.526) | (1.202) | (1.773) |
| **Relationship** | -13.82^**^ | 5.357 | 4.896 | 2.321 | -3.298 | -8.078 |
|  | (6.116) | (6.387) | (3.809) | (5.915) | (5.521) | (6.614) |
| **Offspring** | -6.370 | -12.62 | 3.740 | 0.317 | -4.453 | -5.801 |
|  | (4.800) | (8.656) | (3.220) | (6.879) | (5.074) | (8.250) |
| **Height (cm)** | 0.782^**^ | -0.512 | -0.409^*^ | 0.325 | 0.626^**^ | 0.344 |
|  | (0.320) | (0.329) | (0.208) | (0.414) | (0.312) | (0.327) |
| **Weight (kg)** | -0.0619 | -0.0160 | 0.125 | -0.0817 | -0.124 | 0.0668 |
|  | (0.118) | (0.0981) | (0.103) | (0.0735) | (0.126) | (0.0972) |
| **Breast size** | 0.0776 | -2.194 | 0.519 | -0.647 | 1.113 | -3.101^**^ |
|  | (1.443) | (1.770) | (0.877) | (1.984) | (1.458) | (1.502) |
| **Happiness** | 0.270^*^ | -0.0224 | -0.171 | 0.235 | -0.0811 | 0.0695 |
|  | (0.144) | (0.289) | (0.122) | (0.199) | (0.194) | (0.201) |
| **Health** | -0.243^*^ | -0.205 | 0.158 | -0.169 | -0.0781 | 0.0295 |
|  | (0.143) | (0.223) | (0.117) | (0.203) | (0.137) | (0.208) |
| ***Big 5 personality traits*** |  |  |  |  |  |  |
| **Extraversion** | -2.245 | -3.307 | 0.547 | 2.025 | -1.704 | 1.545 |
|  | (2.763) | (2.135) | (1.422) | (1.788) | (2.526) | (2.034) |
| **Agreeableness** | 3.713 | 4.053 | -1.795 | 0.427 | 4.704 | -5.998^*^ |
|  | (3.561) | (3.998) | (2.275) | (4.109) | (3.302) | (3.346) |
| **Conscientiousness** | 0.817 | 5.998 | 0.565 | -1.191 | -5.393^**^ | -2.223 |
|  | (2.312) | (3.905) | (1.849) | (2.898) | (2.267) | (2.701) |
| **Emotional Stability** | 0.832 | -0.525 | -0.953 | -6.160 | -0.148 | 2.718 |
|  | (2.889) | (5.123) | (1.359) | (4.292) | (2.538) | (3.564) |
| **Openness** | 1.869 | -1.656 | -2.048 | 1.467 | -0.960 | 3.593 |
|  | (2.535) | (2.814) | (1.403) | (4.371) | (2.605) | (3.013) |
| **SOI-R** | -2.844 | 0.735 | 0.505 | 1.501 | 0.277 | -1.275 |
|  | (1.778) | (1.587) | (0.835) | (1.496) | (1.647) | (1.665) |
| **Female surgeon** | -7.601 | 5.104 | 5.729^**^ | 1.363 | 5.134 | 1.402 |
|  | (4.587) | (5.515) | (2.499) | (5.452) | (4.997) | (5.481) |
| ***Prior to video*** |  |  |  |  |  |  |
| **Perceived risk** | 0.383^***^ | 0.543^***^ |  |  |  |  |
|  | (0.0901) | (0.122) |  |  |  |  |
| **Likelihood to recommend** |  |  | 0.766^***^ | 0.639^***^ |  |  |
|  |  |  | (0.0492) | (0.128) |  |  |
| **Likelihood of future surgery** |  |  |  |  | 0.500^***^ | 0.655^***^ |
|  |  |  |  |  | (0.0927) | (0.0909) |
| Constant | -62.31 | 139.5^**^ | 54.17 | -43.51 | -9.932 | -2.456 |
|  | (67.75) | (54.79) | (34.27) | (68.11) | (54.38) | (63.61) |
| N | 93 | 81 | 93 | 81 | 93 | 81 |
| *R^2^* | 0.3516 | 0.4171 | 0.8329 | 0.6636 | 0.4875 | 0.5660 |
| Prob. > *F* | 0.0000 | 0.0000 | 0.0000 | 0.0000 | 0.0000 | 0.0000 |

Notes: OLS coefficient estimates. Standard errors (robust) in parentheses. * *p* < .10; ** *p* < .05; *** *p* < .01.
